# A Radicular Cyst in the Mandibular Anterior Region of Primary Teeth

**DOI:** 10.7759/cureus.63782

**Published:** 2024-07-03

**Authors:** Misa Ishiyama, Shunsuke Namaki, Masako Yaoita, Takashi Kikuiri

**Affiliations:** 1 Department of Pediatric Dentistry, Nihon University School of Dentistry, Tokyo, JPN; 2 Department of Oral and Maxillofacial Surgery, Nihon University School of Dentistry, Tokyo, JPN

**Keywords:** deciduous dentition, mandibular anterior region, case report, primary teeth, radicular cyst

## Abstract

Radicular cysts are common among odontogenic cysts but are rare in primary teeth. They occur more frequently in the mandibular molar region and rarely in the mandibular anterior region. This is a case of a radicular cyst in the mandibular anterior teeth during the primary dentition stage. In addition, after the fenestration of cystic lesions, gauze is generally changed to prevent the extraction socket from closing. However, in this case, the patient was six years old. Therefore, to alleviate the burden of changing the dressing gauze, we practiced putting on and taking off the obturator before fenestration and using it immediately after surgery under general anesthesia. This made it possible to change the dressing gauze after surgery. We were able to maintain an open wound without the burden of dressing gauze changes. The use of the device was shown to be effective in maintaining open wounds in young patients.

## Introduction

Radicular cysts are common among odontogenic cysts and originate from remnants of Malassez epithelial cells of the periodontal ligament in response to inflammation and subsequent tissue necrosis of the dead pulp [1]. According to some reports, the prevalence of radicular cysts in the permanent dentition ranges from 7% to 54%, whereas radicular cysts in the deciduous dentition account for only 0.5% to 3.3% of the total [1,2,3]. Furthermore, most cases occurring in the deciduous dentition are associated with the mandibular molars [3]. The occurrence of radicular cysts in the mandibular anterior region of the deciduous dentition is rare. When a radicular cyst of a deciduous tooth incorporates a permanent tooth germ into itself, and the viability of the tooth germ is weakened, enamel hypoplasia of the permanent tooth occurs. Herein, we describe a case of a radicular cyst in the mandibular anterior tooth during the deciduous period. After diagnosis of the cyst, early cyst fenestration allowed successful induction of permanent tooth eruption, suggesting that proper diagnosis and timing of treatment play an important role in good outcomes.

## Case presentation

**Figure 1:**
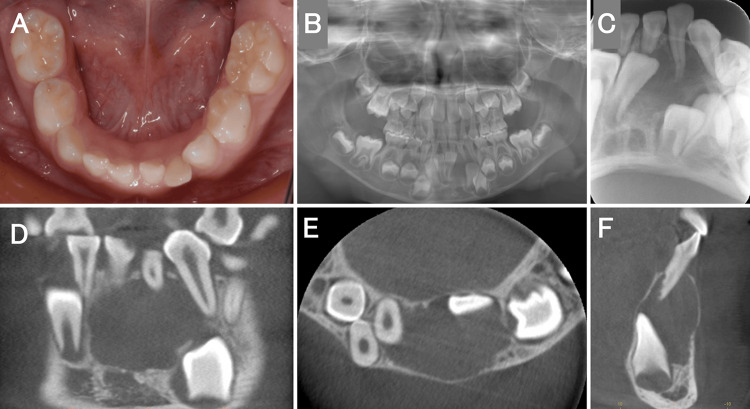
Intraoral photograph and radiographs. (A) Intraoral photograph at the first medical examination. The deciduous canine on the left side of the lower jaw is tilted labially. (B and C) Dental and panoramic photographs at the first medical examination. (D-F) Dental cone-beam computed tomography images at the first medical examination. The low-density image reveals the root apex of the left mandibular deciduous lateral incisor, which is observed to be in contact with the roots of the left mandibular deciduous central incisor and canine.

**Figure 2:**
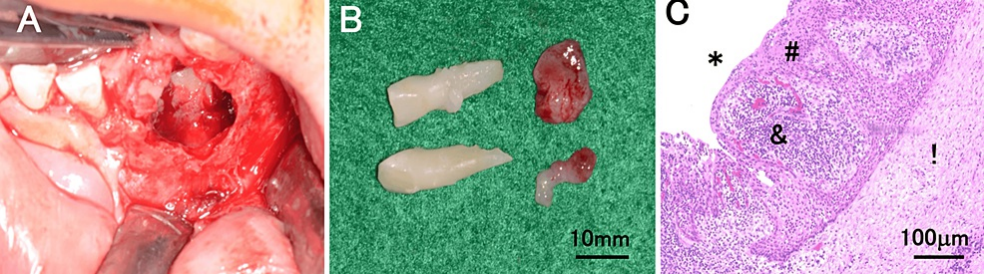
Intraoral photograph after extraction and histopathological images. (A) Intraoral photos during treatment. (B) Extracted deciduous central and lateral incisors along with the cyst. (C) Hematoxylin-and-eosin-stained histological sections. The inner surface of the cyst cavity (*) is lined with keratinizing stratified squamous epithelium (#), and beneath the epithelium is granulation tissue (&) consisting of inflammatory cell infiltration mainly consisting of lymphocytes and plasma cells, proliferation of capillaries, and proliferation of fibroblasts, and further outside of that is a fibrous connective tissue layer (!).

**Figure 3:**
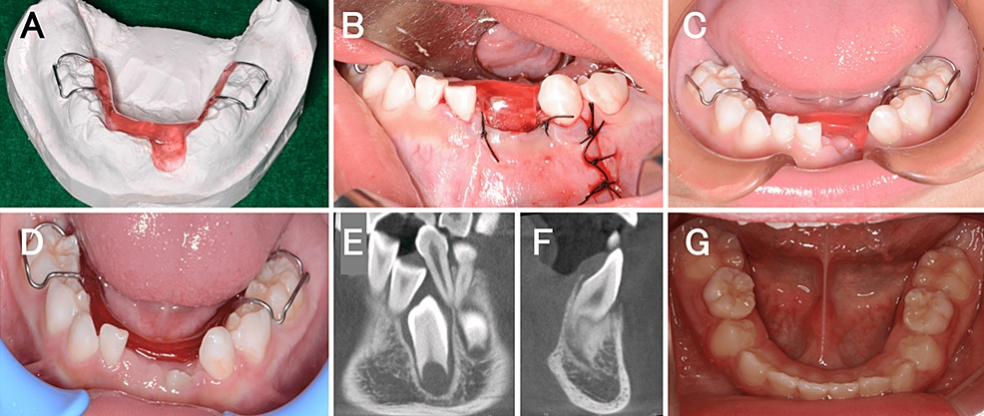
Treatment process. (A and B) An extended obturator to prevent premature closure of the extraction socket. (C) Intraoral photo 14 days after surgery. (D) Intraoral photo one month after surgery. (E and F) Dental cone-beam computed tomography images one year after surgery. (G) Intraoral photo four years after surgery.

The patient was a six-year-old boy who had visited a local doctor for a follow-up, four months back, because of discomfort in the left mandibular anterior tooth. However, he noticed swelling on the lingual side of his left mandibular anterior tooth two weeks prior, which led him to visit our department. Intraoral findings showed that the left mandibular deciduous central incisor was tilted labially (Figure 1A). There was no history of injury. Dental and panoramic radiographic findings showed that the primary lower left central incisor was displaced from the midline to the right and was impacted, with obvious root resorption (Figures 1B-1C). Furthermore, the left mandibular lateral incisor was displaced distally downward. Dental cone beam computed tomography showed an internally uniform diameter of approximately 16 mm from the right mandibular central incisor to the left mandibular canine, with unclear margins from the alveolar crest to the lower mandibular teeth (Figures 1D-1F). However, low-density images revealed that the root apex of the left mandibular deciduous lateral incisor was in contact with the roots of the left mandibular deciduous central incisor and canine. It also showed the crowns of the left mandibular central and lateral incisors, with the latter in contact with the incisal edge of the left mandibular canine. The lingual and labial cortical bones were bulged and thinned, with blurred outlines (Figures 1D-1F). A clinical diagnosis of a dentigerous cyst in the left mandibular lateral incisor was made. Consequently, under infiltration anesthesia, the left mandibular deciduous central and lateral incisors were extracted. The labial alveolar bone was removed, and the cyst was visualized. To remove the cyst, the cyst was peeled off, and the tissues surrounding the left mandibular central and lateral incisors within the cyst were excised using a scalpel (Figure 2A). The extracted soft tissues measured 9 mm × 5 mm × 6 mm and 7 mm × 4 mm × 3 mm (Figure 2B). Histopathological findings showed that the cyst wall was partially hyperemic with granulation and fibrous connective tissues (Figure 2C). The histopathological diagnosis was a radicular cyst. After the cyst was removed, an obturator with an extension was placed in the fenestration to prevent premature closure of the extraction socket (Figures 3A-3B). The obturator was designed to invaginate into the extraction socket. To smoothly use the device immediately after surgery, the patient practiced wearing it with another obturator without an extension before surgery. The device was worn for 14 days, except when washing it after meals. Fourteen days after surgery, the left mandibular central incisor was found to have erupted from the extraction socket, and the device was trimmed (Figure 3C). One month after surgery, eruption of the left mandibular central incisor was observed; therefore, we decided to shave the device and use it as a retainer (Figure 3D). One year after surgery, we observed bilateral central incisors erupting in the mandible (Figures 3E-3F). Subsequently, the mandibular lateral incisor and canine were observed erupting (Figure 3G).

## Discussion

A cyst is a clearly demarcated lesion consisting of an epithelial sac and can be divided into soft tissue and bone cysts, depending on the site of occurrence, with many occurring in the jawbone in the stomatognathic region. Odontogenic epithelium, which normally disappears during tooth eruption, causes cyst formation. The presence of salivary glands, which develop when the epithelium invaginates through the mesenchymal tissue, is also a factor in cyst development. According to the World Health Organization, dentigerous cysts are classified as developmental cysts, which are odontogenic among jawbone cysts. The treatment methods for cysts include fenestration, the Partch I method (marsupialization), the Partch II method (enucleation and primary closure), and open cyst removal (packed open method). The basic treatment is enucleation, however, if enlarged cysts are removed, the surgical intervention can become more invasive, giving rise to concerns about complications [3] such as cortical bone disappearance [4], the cyst being in contact with the mandibular canal [5], or the risk of a pathological fracture. Furthermore, since it is desirable to preserve an adjacent impacted tooth, a two-step treatment may be performed in which fenestration is first performed, and the cyst cavity is then removed after it has shrunk [4]. Fenestration is generally the treatment of choice for children and is considered useful because it preserves permanent teeth, is less invasive as a simpler procedure, and has a good prognosis [6]. In this case, fenestration was chosen because of the patient's age, lesion size, and the goal of preserving the permanent teeth. After the fenestration of a cystic lesion, the gauze is typically replaced to prevent the extraction socket from closing. However, in this case, considering the burden of changing the dressing gauze for our minor patient, we practiced placing and removing the obturator before fenestration. Moreover, by using the device immediately after surgery under general anesthesia, we were able to replace the gauze after surgery. It is possible to maintain an open wound without the burden of replacement. Although the period of use of this obturator was short, it alleviated the burden on the patient. In addition, the ability to use the device as a retainer played an important role in securing the eruption space. Dentigerous cysts generally resemble radicular cysts in X-ray appearance, making it difficult to differentiate between them based on X-ray and clinical findings. Dentigerous cysts are caused by cyst formation in the enamel organ during tooth germ development. In contrast, radicular cysts are generally believed to arise when inflammation caused by pulp necrosis stimulates and proliferates the epithelial rests of Malassez. In the present case, a vitality test of the dental pulp was not performed before surgery. However, the mandibular left deciduous lateral incisor, which is suspected to be the cause of the cyst, did not exhibit signs of caries reaching the dental pulp (Figures 1A-2B). The CT image indicated that the lingual side of the root exhibited an irregular surface and signs of inflammatory root resorption, highly suggesting that it was a nonvital tooth (Figure 1F). The cause of the pulp necrosis of the left mandibular deciduous lateral incisor is unknown. However, the tooth has been displaced to the labial side, suggesting a history of trauma or a worn-down incisal edge. Moreover, the CT image indicates that the pulp cavity has reached the incisal edge, suggesting pulp exposure (Figure 1F). Pathological findings indicated inflammatory cell infiltration in the cyst wall, suggesting that the cyst was caused by an apical lesion of the left mandibular deciduous lateral incisor. Lustman and Shear outlined the following three conditions as diagnostic criteria for radicular cysts of deciduous teeth [7]: (1) the presence of a nonvital deciduous tooth closely associated with a radiolucent lesion; (2) the presence of radicular cyst epithelium in the affected area; and (3) lack of a permanent tooth crown in the cyst cavity. In the present case, the deciduous anterior tooth, which was suspected to be the cause of the cyst, was highly likely to have been a nonvital tooth, as evidenced by the surgical findings indicating continuity between the cyst and the root of the deciduous tooth. Based on these findings, along with the histopathological findings, the diagnosis was a radicular cyst. The lesions are usually noticed by routine radiographic examination of primary teeth endodontic problems while some long-standing lesions may cause appreciable expansion of the cortical bone and display clinical signs and symptoms like swelling, tooth mobility, and displacement of a successful tooth. Radicular cysts often occur in association with endodontically treated primary teeth, so close follow-up is required after endodontic treatment. However, there are also cases where no endodontic treatment has been performed, as in this case, and no history of injury. In such cases, the lesion is discovered after some symptoms have developed, but it is necessary to diagnose and treat it promptly after the symptoms appear.

## Conclusions

Radicular cysts are often asymptomatic, so they are often discovered late. In this study, we performed cyst fenestration immediately after the cyst diagnosis and successfully induced the eruption of the permanent tooth. In addition, because the patient was young, we practiced the placement and removal of the obturator before fenestration and used the device immediately after surgery, which allowed us to maintain an open wound without changing the dressing gauze after surgery. Diagnosis and treatment in cases of radicular cysts associated with deciduous teeth affect the eruption of permanent teeth. As in this case, early diagnosis and appropriate treatment can be expected to lead to a natural eruption of permanent teeth.
